# Supplementary material for: Excessive reactive oxygen species induce transcription-dependent replication stress
Source: Nat Commun. 2023 Mar 30;14:1791. doi: 10.1038/s41467-023-37341-y (PMC10063555; doi:10.1038/s41467-023-37341-y)
Supplement: Supplementary file 1 — Supplementary Information [file 41467_2023_37341_MOESM1_ESM.pdf]

Excessive reactive oxygen species induce transcription-dependent replication stress

Martin Andrs, Henriette Stoy, Barbora Boleslavska, Nagaraja Chappidi, Radhakrishnan Kanagaraj, Zuzana Nascakova, Shruti Menon, Satyajeet Rao, Anna Oravetzova, Jana Dobrovolna, Kalpana Surendranath, Massimo Lopes and Pavel Janscak

Supplementary information

Supplementary Figures 1-7

Supplementary Fig. 1

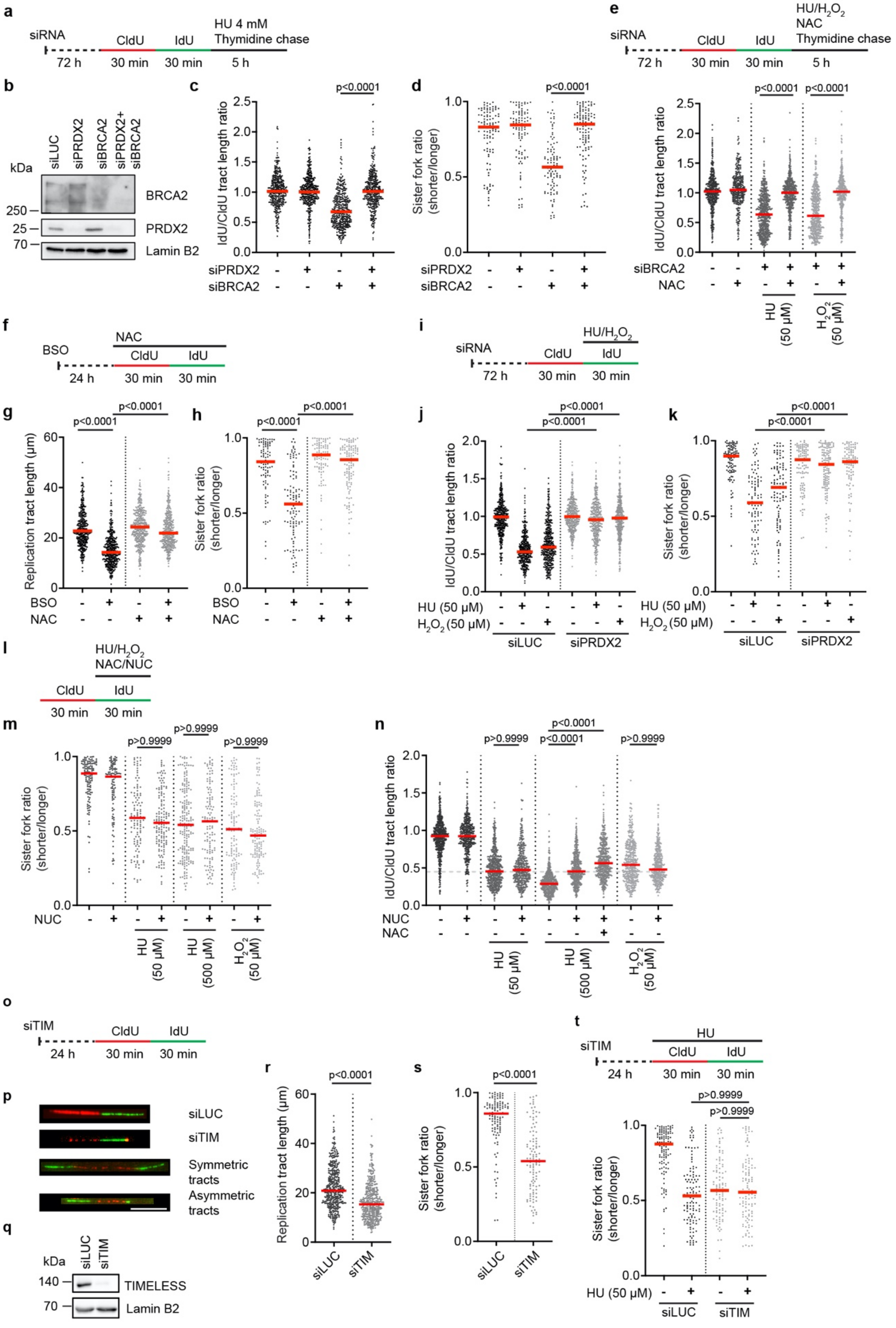

Supplementary Fig. 1. ROS-induced replication slowdown causes replication fork stalling.

a-d PRDX2 depletion prevents nascent DNA degradation in BRCA2-depleted U2OS cells upon exposure to 4 mM hydroxyurea (HU). **a** Experimental workflow of DNA fiber assays. A thymidine (400 μM) chase was included for all conditions to stop IdU incorporation. **b** Western blot analysis of the extracts of U2OS cells transfected with indicated siRNAs. **c** Scatter plot of the values of IdU/CldU tract length ratio obtained from two independent experiments for indicated conditions ($n \geq 406$). **d** Scatter plot of the values of sister IdU tract length ratio obtained from two independent experiments for indicated conditions ($n \geq 100$). **e** Elimination of ROS attenuates nascent DNA strand degradation in BRCA2-depleted U2OS cells upon exposure to 50 μM HU or H_2O_2. *Top*: Experimental workflow of DNA fiber assays. NAC, *N*-acetyl cysteine (5 mM). *Bottom*: Scatter plot of the values of IdU/CldU tract length ratio obtained from two independent experiments for indicated conditions ($n \geq 313$). **f-h** Buthionine sulfoximine (BSO, 10 μM) induces replication fork stalling in U2OS cells in a manner dependent on ROS. **i-k** PRDX2 depletion prevents replication fork stalling induced by 50 μM HU or H_2O_2 in U2OS cells. **f**, **i** Experimental workflow of DNA fiber assays. **g** Scatter plot of the values of replication tract length (IdU + CldU) obtained from two independent experiments for indicated conditions ($n \geq 407$). **j** Scatter plot of the values of IdU/CldU tract length ratio obtained from two independent experiments for indicated conditions ($n \geq 395$). **h**, **k** Scatter plot of the values of sister fork ratio obtained from two independent experiments for indicated conditions ($n \geq 93$). **l-m** Addition of exogenous nucleosides does not rescue HU- or H_2O_2-induced replication fork stalling in U2OS cells. **l** Experimental workflow of DNA fiber assays. NUC, exogenous nucleosides (20 μM each). **m** Scatter plot of the values of sister fork ratio obtained from three independent experiments for indicated conditions ($n \geq 102$). **n** Scatter plot of the values of IdU/CldU tract length ratio obtained from three independent experiments for indicated conditions ($n \geq 405$). **o-s** TIMELESS depletion induces replication fork stalling in U2OS cells. **o** Experimental workflow. **p** Representative images of DNA replication tracts. Scale bar, 10 μm. **q** Western blot analysis of the extracts of U2OS cells transfected with control siRNA (siLuc) and siRNA to TIMELESS (siTIM; 10 nM). **r** Scatter plot of the values of replication tract length (IdU + CldU) obtained from three independent experiments for indicated conditions ($n \geq 424$). **s**, Scatter plot of the values of sister fork ratio obtained from three independent experiments for indicated conditions ($n \geq 113$). **t** HU does not further exacerbate replication fork stalling in TIMELESS-depleted U2OS cells. *Top*: Experimental workflow. *Bottom*: Scatter plot of the values of sister fork ratio obtained from two independent experiments for indicated conditions ($n \geq 100$). **c-e**, **g**, **h**, **j**, **k**, **m**, **n**, **r-t** Red horizontal lines indicate the median; p-values were calculated by Kruskal-Wallis test followed by Dunn's multiple comparisons test. Source data are provided as a Source Data file.

Supplementary Fig. 2

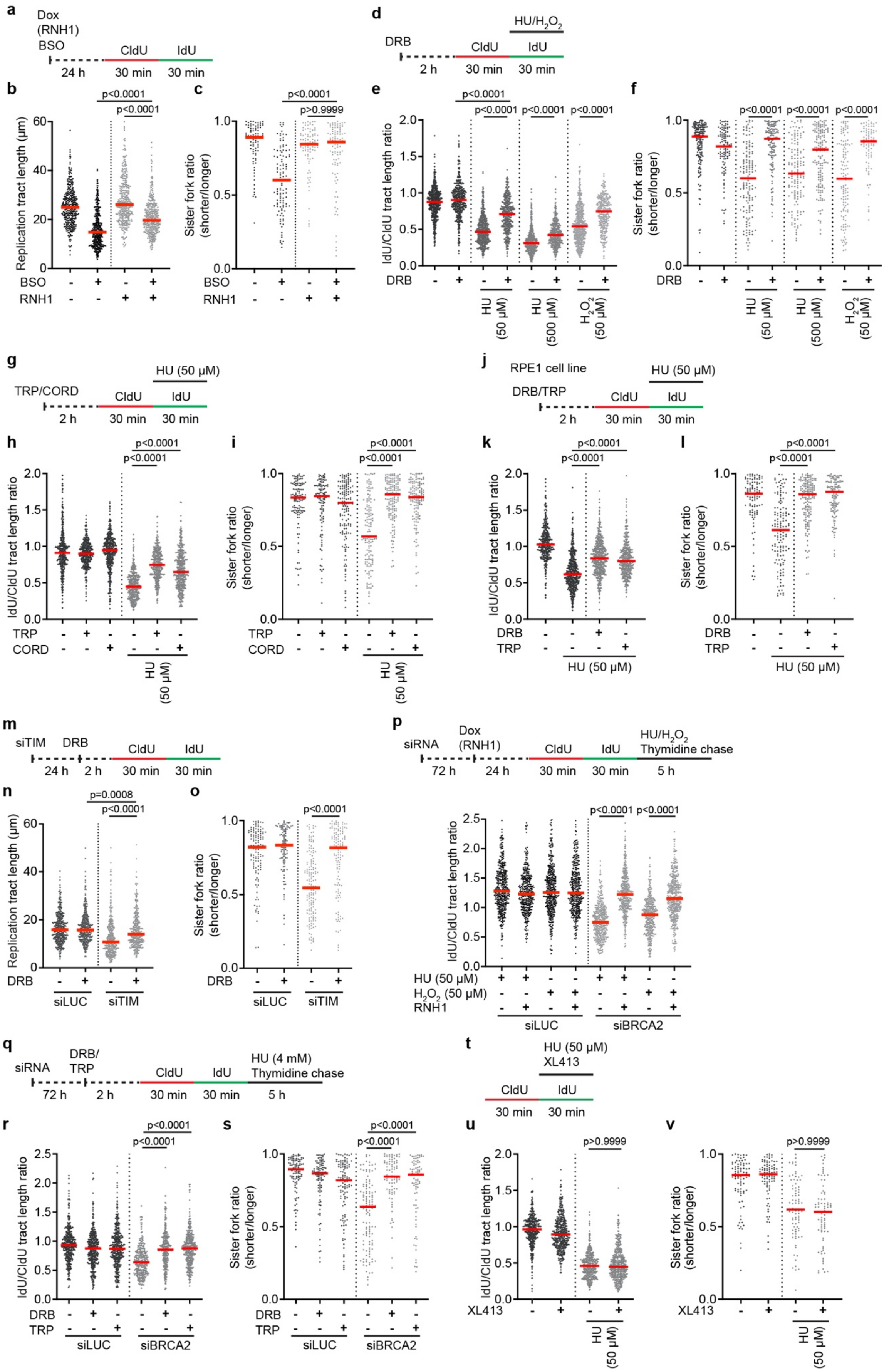

Supplementary Fig. 2. ROS-induced fork stalling is caused by co-transcriptional R-loops.

a-c Overexpression of RNase H1 prevents replication fork stalling induced by buthionine sulfoximine (BSO, 10 μM). **a** Experimental workflow of DNA fiber assays with U2OS T-REx [RNH1(WT)-GFP] cells. Doxycycline (Dox; 1ng/ml) was added to induce RNase H1 (RNH1) expression. **b** Scatter plot of the values of replication tract length (IdU + CldU) obtained from two independent experiments for indicated conditions ($n \geq 406$). **c** Scatter plot of the values of sister fork ratio obtained from two independent experiments for indicated conditions ($n \geq 90$). **d-l** HU- and H_2O_2-induced fork stalling depends on transcription in U2OS (**d-i**) and RPE1 (**j-l**) cells. **d**, **g**, **j** Experimental workflow of DNA fiber assays. DRB, 5,6-dichloro-1-β-D-ribofuranosylbenzimidazole (transcription elongation inhibitor; 100 μM); TRP, triptolide (transcription initiation inhibitor; 1 μM); CORD, cordycepin (transcription elongation inhibitor; 50 μM). **e**, **h**, **k** Scatter plot of the values of IdU/CldU tract length ratio obtained from two independent experiments for indicated conditions ($n \geq 372$). **f**, **i**, **l** Scatter plot of the values of sister fork ratio obtained from two independent experiments for indicated conditions ($n \geq 86$). **m-o** Replication fork stalling induced by TIMELESS depletion depends on transcription. **m** Experimental workflow. U2OS cells were transfected with control siRNA (siLuc) or siRNA to TIMELESS (siTIM; 10 nM). **n** Scatter plot of the values of replication tract length (IdU + CldU) obtained from two independent experiments for indicated conditions ($n \geq 374$). **o** Scatter plot of the values of sister fork ratio obtained from two independent experiments for indicated conditions ($n \geq 106$). **p** RNase H1 overexpression prevents nascent DNA degradation in BRCA2-depleted U2OS cells upon exposure to 50 μM HU or H_2O_2. *Top*: Experimental workflow of DNA fiber assays with U2OS T-REx [RNH1(WT)-GFP] cells. A thymidine (400 μM) chase was included for all conditions to stop IdU incorporation. *Bottom*: Scatter plot of the values of IdU/CldU tract length ratio obtained from two independent experiments for indicated conditions ($n \geq 392$). **q-s** HU-induced nascent DNA degradation in BRCA2-depleted U2OS depends on transcription. **q** Experimental workflow. **r** Scatter plot of the values of IdU/CldU tract length ratio obtained from two independent experiments for indicated conditions ($n \geq 286$). **s** Scatter plot of the values of sister fork ratio obtained from two independent experiments for indicated conditions ($n \geq 81$). **t-v** Inhibition of origin firing does not affect HU-induced fork stalling in U2OS cells. **t** Experimental workflow. XL413, CDC7 inhibitor (2 μM). **u** Scatter plot of the values IdU/CldU tract length ratio obtained from three independent experiments for indicated conditions ($n \geq 399$). **v** Scatter plot of the values of sister fork ratio obtained from three independent experiments for indicated conditions ($n \geq 79$). **b**, **c**, **e**, **f**, **h**, **i**, **k**, **l**, **n**, **o**, **p**, **r**, **s**, **u**, **v** Red horizontal lines indicate the median; p-values were calculated by Kruskal-Wallis test followed by Dunn's multiple comparisons test. Source data are provided as a Source Data file.

Supplementary Fig. 3

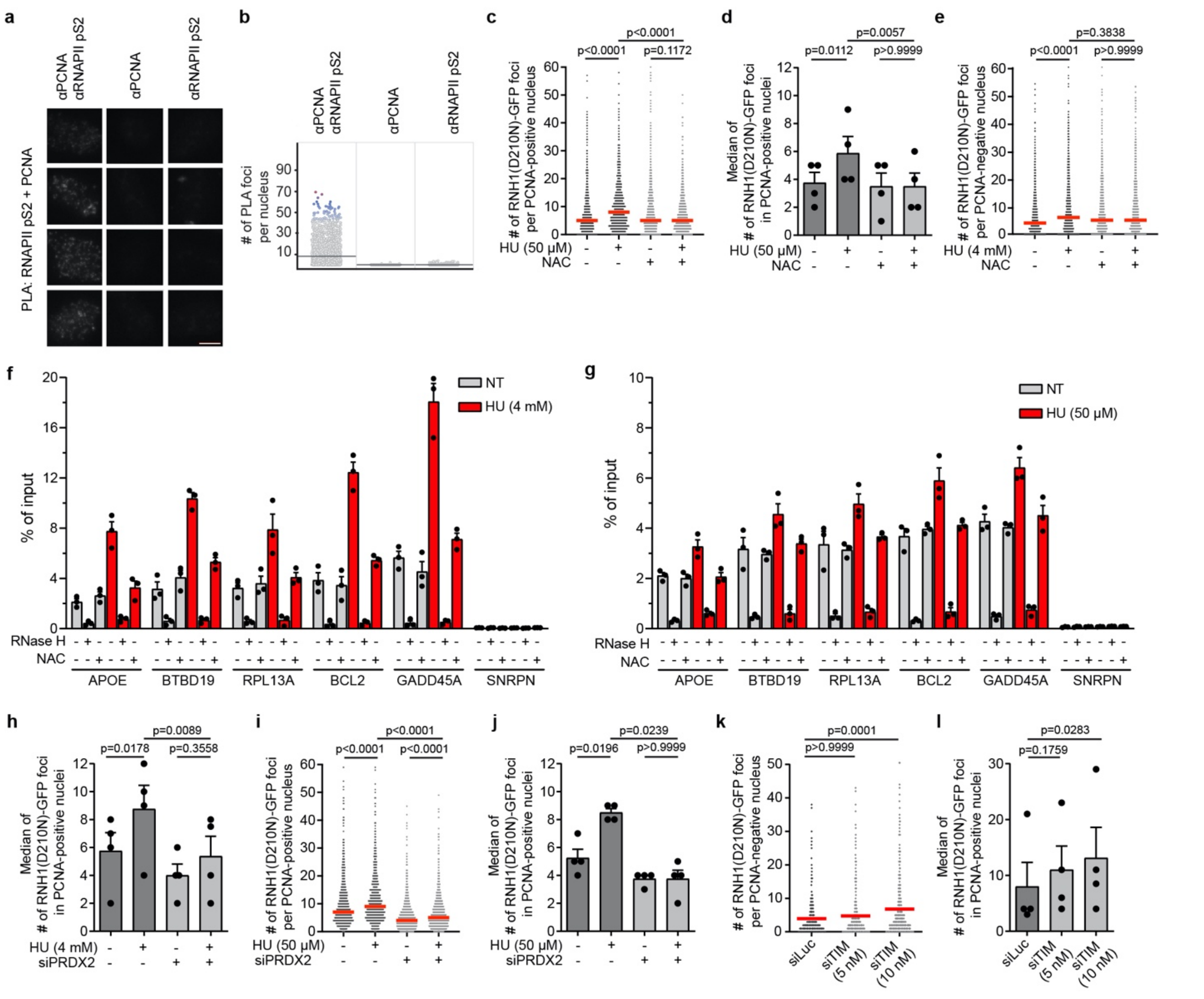

Supplementary Fig. 3. ROS induce transcription-replication conflicts and R-loop formation in a manner dependent on PRDX2.

a, **b** Controls for proximity ligation assay (PLA) to detect colocalization between PCNA and elongating form of RNAPII in U2OS cells. **a** Representative galleries of PLA signal in cell nuclei obtained for single antibodies (PCNA and RNAPII pS2) and their combination. Images were exported from the ScanR analysis program. Scale bar, 10 µm. **b** Scatter plot of PLA foci numbers. Grey horizontal lines indicate the median (n ≥ 1508). A representative plot from three independent experiments yielding similar results is shown. **c** Scatter plot of the number of RNH1(D210N)-GFP foci in PCNA-positive nuclei for indicated conditions (n ≥ 903). A representative plot from four independent experiments yielding similar results is shown. **d** Plot of the median values of the data sets represented in **c** (n = 4). **e** Scatter plot of the number of RNH1(D210N)-GFP foci in PCNA-negative nuclei of U2OS T-REx [RNH1(D210N)-GFP] cells measured for indicated conditions (n ≥

1115); linked to Fig. 3d-g. **f**, **g** ROS induce accumulation of RNA:DNA hybrids at R-loop-prone loci in U2OS cells. Cells were treated with 4 mM HU (**f**) or 50 μM HU (**g**) for 1 h. Where indicated, NAC (5 mM) was present during the treatment. Isolated genomic DNA was subjected to DRIP-qPCR analysis. An R-loop-free *SNRPN* gene locus was used as a negative control. Where indicated, the genomic DNA was treated with RNase H to remove RNA:DNA hybrids. Data are plotted as a percentage of the input, and represent the mean ± SEM, n = 3. NT, non-treated. **h** Plot of the median values of the data sets represented in Fig. 3h (n = 4). **i** Scatter plot of the number of RNH1(D210N)-GFP foci in PCNA-positive nuclei for indicated conditions (n ≥ 910). A representative plot from four independent experiments yielding similar results is shown. **j** Plot of the median values of the data sets represented in **i**. **k** Scatter plot of the number of RNH1(D210N)-GFP foci in PCNA-negative nuclei of U2OS T-REx [RNH1(D210N)-GFP] cells transfected with control siRNA (siLuc) and siRNA to TIMELESS (siTIM) (n ≥ 344); linked to Fig. 3j,k. **l** Plot of the median values of the data sets represented in Fig. 3j (n = 4). **c**, **e**, **i**, **k** Red horizontal lines indicate the median; p-values were calculated by Kruskal-Wallis test followed by Dunn's multiple comparisons test. **d**, **h**, **j**, **l** Data are presented as mean ± SEM; p-values were calculated by one-way ANOVA followed by Tukey's test. Source data are provided as a Source Data file.

Supplementary Fig. 4

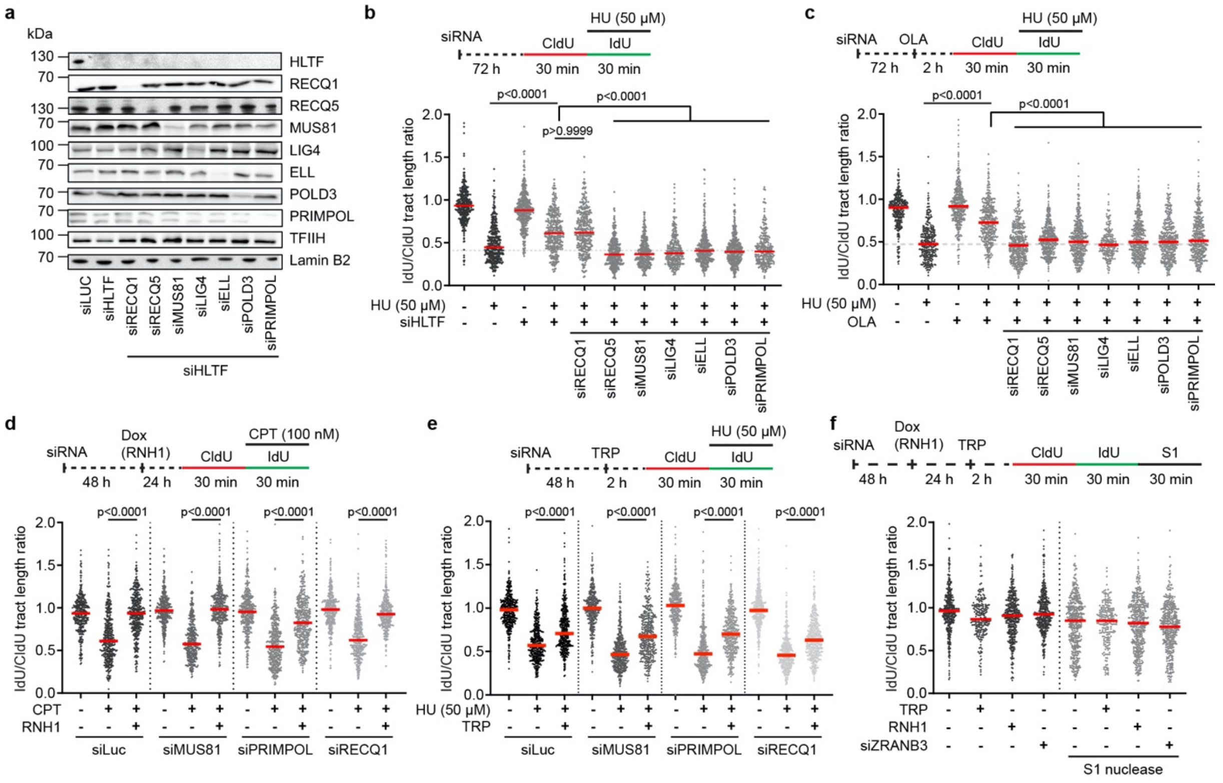

Supplementary Fig. 4. MUS81-LIG4-PRIMPOL axis mediates replication restart following ROS-induced fork stalling.

a Western blot analysis of the extracts of U2OS cells transfected with indicated siRNAs. **b** HLTF depletion restores replication fork progression in HU-treated U2OS cells in a manner dependent on PRIMPOL and the proteins required for restarting R-loop-stalled forks. *Top*: Experimental workflow of DNA fiber assays. *Bottom*: Scatter plot of the values of IdU/CldU tract length ratio obtained from two independent experiments for indicated conditions (n ≥ 266). **c** PARP inhibition with olaparib restores replication fork progression in HU-treated U2OS cells in a manner dependent on the same set of proteins as in **b**. *Top*: Experimental workflow. OLA, olaparib (10 μM). *Bottom*: Scatter plot of the values of IdU/CldU tract length ratio obtained from two independent experiments for indicated conditions (n ≥ 216). **d** Rescue of CPT-induced fork slowing by RNase H1 overexpression does not depend upon MUS81, PRIMPOL and RECQ1. *Top*: Experimental workflow. Dox, doxycycline (1 ng/ml), CPT, camptothecin (100 nM). *Bottom*: Scatter plot of the values of IdU/CldU tract length ratio obtained from two independent experiments for indicated conditions (n ≥ 362). **e** Transcription inhibition restores replication fork progression in HU-treated U2OS cells in a manner independent of MUS81 and PRIMPOL. *Top*: Experimental workflow. TRP, triptolide (transcription initiation

inhibitor; 1 μM). *Bottom*: Scatter plot of the values of IdU/CldU tract length ratio obtained from two independent experiments for indicated conditions ($n \geq 399$). **f** DNA fiber assay using S1 nuclease with U2OS T-REx [RNH1(WT)-GFP] cells transfected with control siRNA (siLuc) or siRNA to ZRANB3 (where indicated). *Top*: Experimental workflow. *Bottom*: Scatter plot of the values of IdU/CldU tract length ratio obtained from two independent experiments for indicated conditions ($n \geq 214$). **b**-**f** Red horizontal lines indicate the median; p-values were calculated by Kruskal-Wallis test followed by Dunn's multiple comparisons test. Source data are provided as a Source Data file.

Supplementary Fig. 5

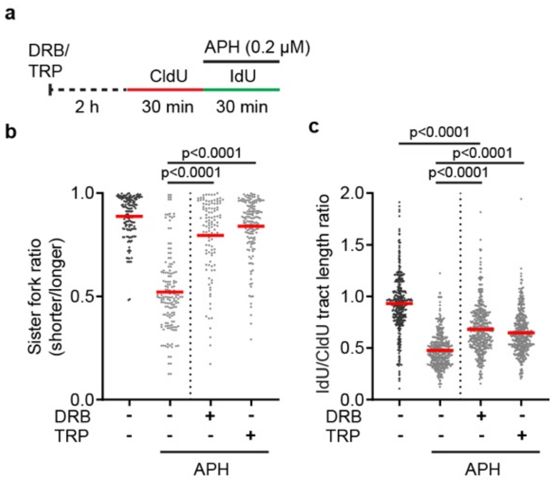

Supplementary Fig. 5. R-loop-dependent fork stalling is induced by partial inhibition of DNA synthesis with aphidicolin.

a-c Inhibition of transcription rescues replication fork stalling induced by low doses of aphidicolin. **a** Experimental workflow of DNA fiber assays with U2OS cells. DRB, 5,6-dichloro-1-β-D-ribofuranosylbenzimidazole (transcription elongation inhibitor; 100 µM); TRP, triptolide (transcription initiation inhibitor; 1 µM). **b** Scatter plot of the values of sister fork ratio obtained from three independent experiments for indicated conditions ($n \geq 112$). **c** Scatter plot of the values of IdU/CldU tract length ratio obtained from three independent experiments for indicated conditions, ($n \geq 375$). **b**, **c** Red horizontal lines indicate the median; p-values were calculated by Kruskal-Wallis test followed by Dunn's multiple comparisons test. Source data are provided as a Source Data file.

Supplementary Fig. 6

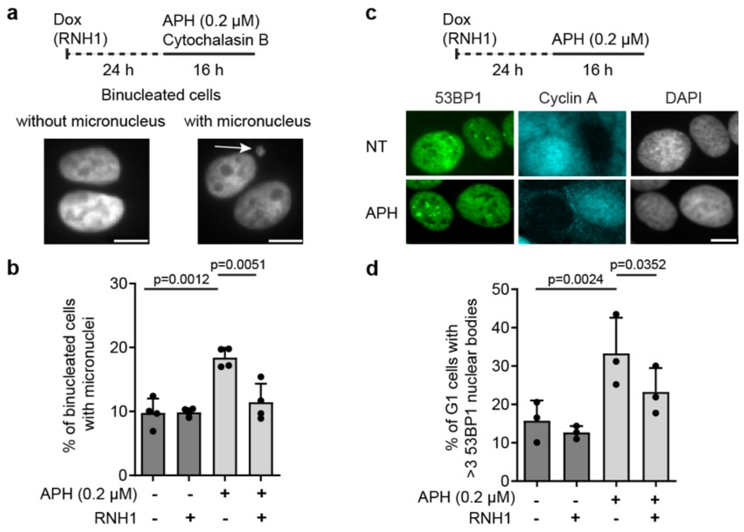

Supplementary Fig. 6. Replication slowdown leads to R-loop-dependent micronucleation and accumulation of 53BP1 nuclear bodies in nascent G1 cells.

a, **b** Aphidicolin (APH) induces R-loop-dependent micronucleation. **a** *Top*: Experimental workflow. U2OS T-REx [RNH1(WT)-GFP] cells were treated with 0.2 µM APH for 16 h. RNase H1 expression was induced by doxycycline (Dox; 1 ng/ml) 24 h before APH addition. Cytochalasin B (2 µg/ml; 16 h) was added to block cells in cytokinesis. *Bottom*: Representative images of binucleated U2OS T-REx [RNH1(WT)-GFP] cells without (left) or with (right) a micronucleus (white arrow). Scale bar, 10 µm. **b** Quantification of the frequency of micronuclei for indicated conditions. Data are presented as mean ± SD (n = 4); p-values were calculated by one-way ANOVA followed by Tukey's test. For each condition, at least 150 binucleated cells were examined for the presence of micronuclei in each experiment. **c**, **d** APH induces R-loop-dependent formation of 53BP1 nuclear bodies in G1 cells. **c** *Top*: Experimental workflow. *Bottom*: Immunofluorescence images showing examples of G1 cells (cyclin A-negative) containing 53BP1 nuclear bodies. U2OS T-REx [RNH1(WT)-GFP] cells were either left untreated (NT) or treated with 0.2 µM APH for 16 h. Scale bar, 10 µm. **d** Quantification of G1-specific 53BP1 nuclear bodies for indicated conditions. RNase H1 expression was induced 24 h before APH treatment. The percentage of G1 cells with > 3 bodies is plotted. Data are presented as mean ± SD (n = 3); p-values were calculated by one-way ANOVA followed by Tukey's test. For each condition, at least 300 cyclin A-negative cells were analyzed in each experiment to determine the number of 53BP1 nuclear bodies. Source data are provided as a Source Data file.

Supplementary Fig. 7

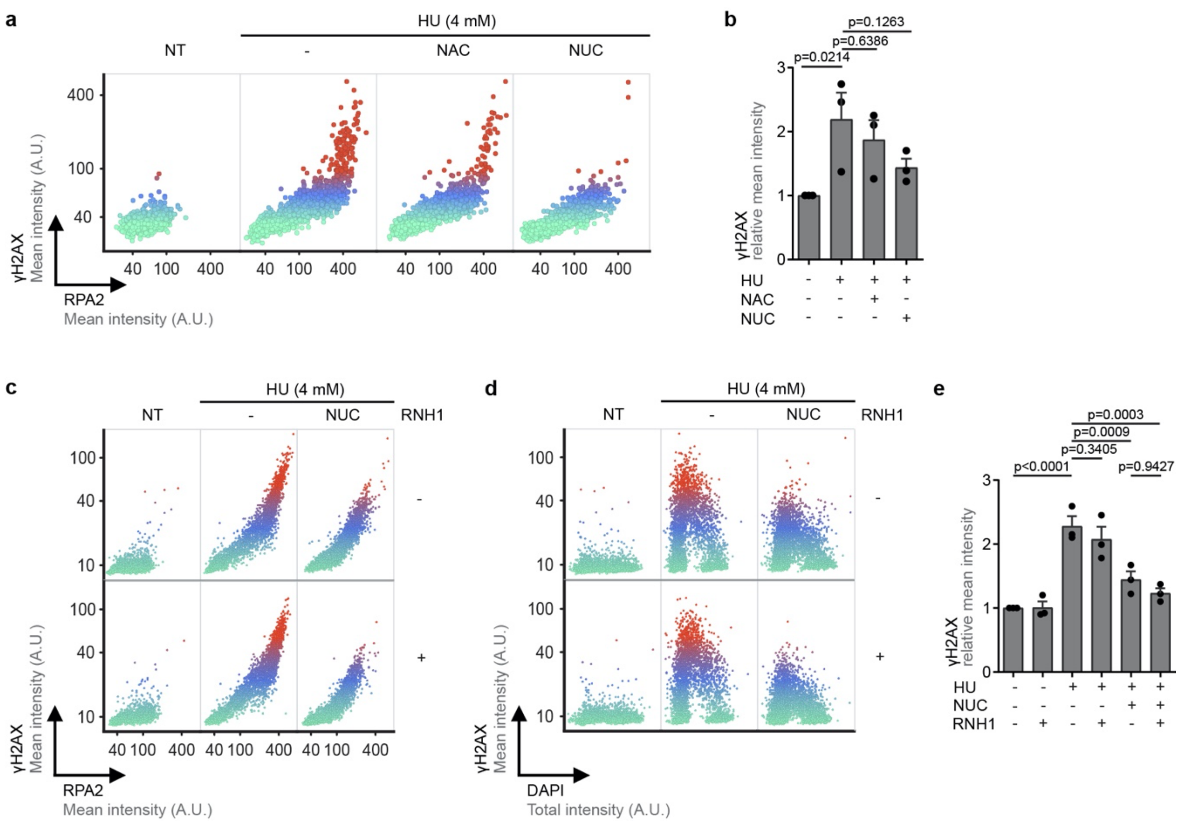

Supplementary Fig. 7. Replication arrest due to dNTP shortage leads to R-loop-independent DNA breakage

a Scatter plots showing the mean intensity of RPA2 (x-axis) versus the mean intensity of γH2AX (y-axis) in individual U2OS cells for indicated conditions, as measured by QIBC. Representative plots from three independent experiments yielding similar results are shown. At least 1000 cells were analyzed for each condition in each experiment. Cells were treated with 4 mM HU for 4 h in the absence or presence of *N*-acetyl cysteine (NAC; 5 mM) or exogenous nucleosides (NUC; 20 µM each). Individual cells are colored based on γH2AX intensity (green: low, blue: moderate, red: high). NT, non-treated; A.U., arbitrary units. **b** Plot of mean intensity of γH2AX from the QIBC analysis represented in **a**. **c**, **d** Scatter plots showing the mean intensity of γH2AX (y-axis) versus the mean intensity of RPA (**c**) or DAPI (**d**) in individual U2OS T-REx [RNH1(WT)-GFP] cells for indicated conditions, as measured by QIBC. Representative plots from three independent experiments yielding similar results are shown. At least 1000 cells were analyzed for each condition in each experiment. RNase H1 (RNH1) expression was induced by doxycycline (1 ng/ml) 24 h before the addition of HU. **e** Plot of mean intensity of γH2AX from the QIBC analysis represented in (**c**). **b**, **e** Data are presented

as mean ± SEM normalized to control (NT); p-values were calculated by one-way ANOVA followed by Tukey's test. Source data are provided as a Source Data file.
