# Supplementary material for: Excessive reactive oxygen species induce transcription-dependent replication stress
Source: Nat Commun. 2023 Mar 30;14:1791. doi: 10.1038/s41467-023-37341-y (PMC10063555; doi:10.1038/s41467-023-37341-y)
Supplement: Supplementary file 3 — Reporting Summary [file 41467_2023_37341_MOESM3_ESM.pdf]

nature portfolio

Corresponding author(s): Pavel Janscak

Last updated by author(s): Feb 20, 2023

Reporting Summary

Nature Portfolio wishes to improve the reproducibility of the work that we publish. This form provides structure for consistency and transparency in reporting. For further information on Nature Portfolio policies, see our Editorial Policies and the Editorial Policy Checklist.

Statistics

For all statistical analyses, confirm that the following items are present in the figure legend, table legend, main text, or Methods section.

n/a	Confirmed	
☐	☒	The exact sample size (*n*) for each experimental group/condition, given as a discrete number and unit of measurement
☐	☒	A statement on whether measurements were taken from distinct samples or whether the same sample was measured repeatedly
☐	☒	The statistical test(s) used AND whether they are one- or two-sided<br>*Only common tests should be described solely by name; describe more complex techniques in the Methods section.*
☒	☐	A description of all covariates tested
☐	☒	A description of any assumptions or corrections, such as tests of normality and adjustment for multiple comparisons
☐	☒	A full description of the statistical parameters including central tendency (e.g. means) or other basic estimates (e.g. regression coefficient) AND variation (e.g. standard deviation) or associated estimates of uncertainty (e.g. confidence intervals)
☐	☒	For null hypothesis testing, the test statistic (e.g. *F*, *t*, *r*) with confidence intervals, effect sizes, degrees of freedom and *P* value noted<br>*Give P values as exact values whenever suitable.*
☒	☐	For Bayesian analysis, information on the choice of priors and Markov chain Monte Carlo settings
☒	☐	For hierarchical and complex designs, identification of the appropriate level for tests and full reporting of outcomes
☒	☐	Estimates of effect sizes (e.g. Cohen's *d*, Pearson's *r*), indicating how they were calculated

Our web collection on statistics for biologists contains articles on many of the points above.

Software and code

Policy information about availability of computer code

Data collection	QIBC data collection was performed using Olympus ScanR acquisition software (version 3.1); other microscopy images were acquired by Leica Application Suite X 3.6.0.20104
Data analysis	QIBC microscopy images were analyzed by Olympus ScanR analysis software (vession 3.0.1 or 3.3.1); DNA fibers and micronuclei were analyzed by ImageJ Fiji software; EM images were analyzed by MAPS software package (ThermoFisher Scientific, Netherlands); data analysis and statistics were done in Graphpad Prism 9 (version 9.4.1) and TIBCO Spotfire (version 7.0.1).

For manuscripts utilizing custom algorithms or software that are central to the research but not yet described in published literature, software must be made available to editors and reviewers. We strongly encourage code deposition in a community repository (e.g. GitHub). See the Nature Portfolio guidelines for submitting code & software for further information.

Data

Policy information about availability of data

All manuscripts must include a data availability statement. This statement should provide the following information, where applicable:

- Accession codes, unique identifiers, or web links for publicly available datasets
- A description of any restrictions on data availability
- For clinical datasets or third party data, please ensure that the statement adheres to our policy

The authors declare that all data supporting the findings of this study are available within the paper. The source data for Figs 1-7 and Supplementary Figs. 1-7 are provided as a Source Data file. All original microscopy images will be made available upon reasonable request.

Human research participants

Policy information about studies involving human research participants and Sex and Gender in Research.

Reporting on sex and gender	N/A
Population characteristics	N/A
Recruitment	N/A
Ethics oversight	N/A

Note that full information on the approval of the study protocol must also be provided in the manuscript.

Field-specific reporting

Please select the one below that is the best fit for your research. If you are not sure, read the appropriate sections before making your selection.

[x] Life sciences [ ] Behavioural & social sciences [ ] Ecological, evolutionary & environmental sciences

For a reference copy of the document with all sections, see nature.com/documents/nr-reporting-summary-flat.pdf

Life sciences study design

All studies must disclose on these points even when the disclosure is negative.

Sample size	Sample size for all experiments shown (electron microscopy, n>70 in 3 independent experiments; DNA fibers, n>150 in 2 or more independent experiments for IdU/CldU ratio or tract lengths, n>40 in 2 or more independent experiments for sister fork asymmetry; micronuclei, n>150 in 3 or more independent experiments, QIBC, >500 in 3 or more independent experiments) was chosen to obtain statistical power, in conformity to accepted standard sample size in a number of previous publications using these approaches:<br>Berti et al., Nat Commun., DOI: 10.1038/s41467-020-17324-z<br>Mijic et al., Nat Commun., DOI: 10.1038/s41467-017-01164-5<br>Vujanovic et al., Mol Cell, DOI: 10.1016/j.molcel.2017.08.010<br>Mutreja et al., Cell Rep., DOI: 10.1016/j.celrep.2018.08.019
Data exclusions	No data were excluded from any of the analyses.
Replication	For all experiments, the number of biological replicates is indicated and, without any exception, reproduced the representative data shown in the figures.
Randomization	Experiments were not randomized. We were working with asynchronously cycling cell populations or individual DNA replication molecules from these cell populations. Hence further randomization or covariates management were not necessary for our approaches.
Blinding	Individual repetitions for DNA fiber analysis, micronuclei analysis and Electron Microscopy were blinded to the investigators.<br>For the automated QIBC screen blinding was not necessary due to its intrinsically unbiased nature.

Reporting for specific materials, systems and methods

We require information from authors about some types of materials, experimental systems and methods used in many studies. Here, indicate whether each material, system or method listed is relevant to your study. If you are not sure if a list item applies to your research, read the appropriate section before selecting a response.

Materials & experimental systems

n/a	Involved in the study
☐	☒ Antibodies
☐	☒ Eukaryotic cell lines
☒	☐ Palaeontology and archaeology
☒	☐ Animals and other organisms
☒	☐ Clinical data
☒	☐ Dual use research of concern

Methods

n/a	Involved in the study
☒	☐ ChIP-seq
☒	☐ Flow cytometry
☒	☐ MRI-based neuroimaging

Antibodies

Antibodies used

Primary antibodies:
BrdU (CldU) rat (ab6326, Abcam), BrdU (IdU) mouse (347580, BD Biosciences), RPA2 (9H8) mouse monoclonal (ab2175, Abcam), γH2AX (S139) mouse monoclonal (05-636, Merck-Millipore), RPA2 pS4/8 rabbit polyclonal (A300-245A, Bethyl Laboratories), PCNA (PC10) mouse monoclonal (sc56, Santa Cruz Biotechnology), 53BP1 rabbit polyclonal (sc22760, Santa Cruz Biotechnology), Cyclin A (B-8) mouse monoclonal (sc-271682, Santa Cruz Biotechnology), PCNA rabbit polyclonal (ab18197, Abcam), RNA polymerase II, H5 mouse monoclonal (920204, BioLegend), MUS81 (MTA30 2G103) mouse monoclonal (sc-53382, Santa Cruz Biotechnology), TFIIH p89 (S-19) rabbit polyclonal (sc-293, Santa Cruz Biotechnology), RECQ5 rabbit polyclonal (Janscak lab, Urban JCB 2016), RECQ1 rabbit polyclonal (NB100-182, Novus Biological), LIG4 (D-8) mouse monoclonal (sc-271299, Santa Cruz Biotechnology), BRCA2 (Ab-1) mouse monoclonal (OP-95, EMD Millipore), RNASE H1 (A-9) mouse monoclonal (sc-365783, Santa Cruz Biotechnology), POLD3 (M01, clone 3E2) mouse monoclonal (H00010714-M01, Abnova), ZRANB3 rabbit polyclonal (23111- 1-AP, Proteintech), ELL (B-4) mouse monoclonal (sc-398959, Santa Cruz Biotechnology), HLTF mouse monoclonal (sc-398357, Santa Cruz Biotechnology), Lamin B2 (gt144) mouse monoclonal (gtx628803, Genetex), TIMELESS (EPR5275) rabbit monoclonal (ab109512, Abcam), PRDX2 mouse monoclonal (sc-515428, Santa Cruz Biotechnology); PRIMPOL rat antibody was kindly provided by Dr. Juan Mendez (Mouron et al., 2013), RNA:DNA hybrid (S9.6) mouse monoclonal (ENH001, Kerafast)
Secondary antibodies:
Alexa Fluor 488 Goat Anti-Rabbit IgG (A110334, Thermo Fisher Scientific), Alexa Fluor 594 Goat Anti-Rabbit IgG (A11037, Thermo Fisher Scientific), Alexa Fluor 488 Goat Anti-Mouse IgG (A11001, Thermo Fisher Scientific), Alexa Fluor 594 Goat Anti-Mouse IgG (A11005, Thermo Fisher Scientific). Alexa Fluor 647 Goat Anti-Mouse IgG (A21235, Thermo Fisher Scientific), donkey anti-rat Cy3 (712-166-153, Jackson ImmunoResearch), goat anti-rabbit IgG-HRP (A0545, Sigma-Aldrich), goat anti-mouse IgG HRP (A4416, Sigma-Aldrich), goat anti-rat IgG HRP (SC2006, Santa Cruz Biotechnology)

Validation

Specificities of the antibodies against MUS81, RECQ5, RECQ1, LIG4, BRCA2, POLD3, ZRANB3, ELL, HLTF, TIMELESS, PRDX2 and PRIMPOL were confirmed by protein knockdown with specific siRNA for the corresponding target. Specificity of the antibody against RNASE H1 was confirmed protein over-expression in U2OS T-REx RNH1-GFP cells.
Specificities of the antibodies against TFIIH and LaminB were validated by the manufacturer for western blots as indicated on manufacturer website (see below). Specificity of the antibodies against RPA2, γH2AX, 53BP1, RPA2 pS4/8, PCNA (PC10), Cyclin A, PCNA (polyclonal) and RNA polymerase II (H5) were validated by the manufacturer for immunofluorescence as indicated on manufacturer website (see below). Specificity of the antibody against RNA:DNA hybrid (S9.6) was validated by Smolka et al, 2021 (DOI: 10.1083/jcb.202004079). The antibodies against BrdU (CldU) and BrdU (IdU) are routinely used for DNA fiber spreading assay.

BrdU (CldU) rat (ab6326, Abcam); https://www.abcam.com/brdu-antibody-bu175-icr1-proliferation-marker-ab6326.html
BrdU (IdU) mouse (347580, BD Biosciences); https://www.bdbiosciences.com/en-eu/products/reagents/flow-cytometry-reagents/clinical-discovery-research/single-color-antibodies-ruo-gmp/purified-mouse-anti-brdu.347580
RPA2 (9H8) mouse monoclonal (ab2175, Abcam); https://www.abcam.com/rpa32rpa2-antibody-9h8-ab2175.html
γH2AX (S139) mouse monoclonal (05-636, Merck-Millipore); https://www.merckmillipore.com/CZ/cs/product/Anti-phospho-Histone-H2A.X-Ser139-Antibody-clone-JBW301,MM_NF-05-636
RPA2 pS4/8 rabbit polyclonal (A300-245A, Abcam); https://www.thermofisher.com/antibody/product/Phospho-RPA32-Ser4-Ser8-Antibody-Polyclonal/A300-245A
PCNA (PC10) mouse monoclonal (sc56, Santa Cruz Biotechnology); https://www.scbt.com/p/pcna-antibody-pc10?requestFrom=search
53BP1 rabbit polyclonal (sc22760, Santa Cruz Biotechnology); https://www.scbt.com/p/53bp1-antibody-h-300?requestFrom=search
Cyclin A (B-8) mouse monoclonal (sc-271682, Santa Cruz Biotechnology); https://www.scbt.com/p/cyclin-a-antibody-b-8
PCNA rabbit polyclonal (ab18197, Abcam); https://www.abcam.com/pcna-antibody-ab18197.html
RNA polymerase II, H5 mouse monoclonal (920204, BioLegend); https://www.biolegend.com/ja-jp/products/purified-anti-rna-polymerase-ii-rpb1-antibody-13077?GroupID=GROUP26
TFIIH p89 (S-19) rabbit polyclonal (sc-293, Santa Cruz Biotechnology); https://www.scbt.com/p/tfiih-p89-antibody-s-19
RNA:DNA hybrid (S9.6) mouse monoclonal (ENH001, Kerafast); https://www.kerafast.com/productgroup/432/anti-dna-rna-hybrid-s96-antibody
Lamin B2 (gt144) mouse monoclonal (gtx628803, Genetex); https://www.genetex.com/Product/Detail/Lamin-B2-antibody-GT144/GTX628803

Eukaryotic cell lines

Policy information about cell lines and Sex and Gender in Research

Cell line source(s)

U2OS (ATCC HTB-96), HeLa Kyoto (ATCC CVCL 1922) and RPE1 (ATCC CRL-4000)
U2OS T-REx RNH1-GFP was prepared in Janscak lab (doi: 10.1016/j.molcel.2019.10.026)

	U2OS T-REx RNH1-D210N-GFP was prepared in Janscak lab (doi: 10.1016/j.molcel.2018.11.036)<br>HeLa Kyoto MUS81 KO was prepared in Joao Matos lab (doi: 10.1016/j.molcel.2019.10.026)
Authentication	None of the cell lines were authenticated in house for this manuscript.
Mycoplasma contamination	We routinely do mycoplasma testing on our cell lines.
Commonly misidentified lines<br>(See ICLAC register)	No commonly misidentified lines were used in this study.
